# Crosstalk between TM4SF5 and GLUT8 regulates fructose metabolism in hepatic steatosis

**DOI:** 10.1016/j.molmet.2022.101451

**Published:** 2022-02-02

**Authors:** Hyejin Lee, Eunmi Kim, Eun-Ae Shin, Jong Cheol Shon, Hyunseung Sun, Ji Eon Kim, Jae Woo Jung, Haesong Lee, Yangie Pinanga, Dae-Geun Song, Kwang-Hyeon Liu, Jung Weon Lee

**Affiliations:** 1 Department of Pharmacy, College of Pharmacy, Seoul National University, Seoul 08826, South Korea; 2 Research Institute of Pharmaceutical Sciences, Seoul National University, Seoul 08826, South Korea; 3 Department of Pharmacy, College of Pharmacy, Kyungpook National University, Daegu 41566, South Korea; 4 Natural Product Informatics Research Center, Korea Institute of Science and Technology (KIST), Gangneung-si, Gangwon-do 25451, South Korea

**Keywords:** Fructose metabolism, Steatosis, Triacylglycerol, GLUT8, Tetraspanin

## Abstract

**Objective:**

Transmembrane 4 L six family member 5 (TM4SF5) is likely involved in non-alcoholic steatohepatitis, although its roles and cross-talks with glucose/fructose transporters in phenotypes derived from high-carbohydrate diets remain unexplored. Here, we investigated the modulation of hepatic fructose metabolism by TM4SF5.

**Methods:**

Wild-type or *Tm4sf5*^−/−^ knockout mice were evaluated via different diets, including normal chow, high-sucrose diet, or high-fat diet without or with fructose in drinking water (30% w/v). Using liver tissues and blood samples from the mice or hepatocytes, the roles of TM4SF5 in fructose-mediated *de novo* lipogenesis (DNL) and steatosis via a crosstalk with glucose transporter 8 (GLUT8) were assessed.

**Results:**

*Tm4sf5* suppression or knockout in both *in vitro* and *in vivo* models reduced fructose uptake, DNL, and steatosis. Extracellular fructose treatment of hepatocytes resulted in an inverse relationship between fructose–uptake activity and TM4SF5-mediated translocalization of GLUT8 through dynamic binding at the cell surface. Following fructose treatment, TM4SF5 binding to GLUT8 transiently decreased with translocation to the plasma membrane (PM), where GLUT8 separated and became active for fructose uptake and DNL.

**Conclusions:**

Overall, hepatic TM4SF5 modulated GLUT8 localization and activity through transient binding, leading to steatosis-related fructose uptake and lipogenesis. Thus, TM4SF5 and/or GLUT8 may be promising treatment targets against liver steatosis resulting from excessive fructose consumption.

## Highlights

• The impact of TM4SF5 in fructose metabolism for nonalcoholic fatty liver disease (NAFLD) is not documented.
• Hepatic TM4SF5 could be associated with fructose-mediated nonalcoholic fatty liver.
• TM4SF5 regulated intracellular localization and fructose uptake activity of GLUT8.
• TM4SF5 inhibitors may attenuate phenotypes of NAFLD by excessive fructose intake.

## Graphical abstract

**Figure:**
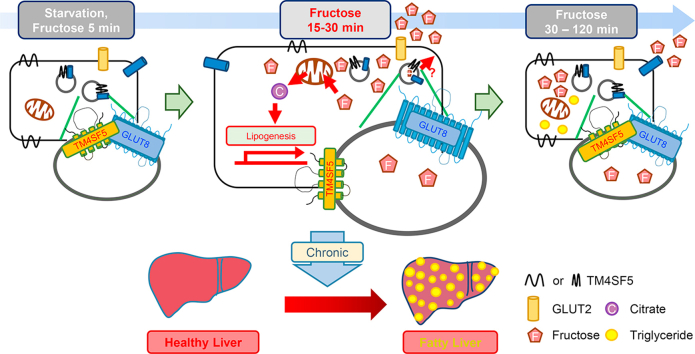

## 1 Introduction

The prevalence of non-alcoholic fatty liver disease (NAFLD) in the population is 25% worldwide, with metabolic dysfunction being a primary cause, in addition to hepatic viral infection and toxin accumulation. Western diets and/or those involving substantial daily consumption of sugars (sucrose, glucose, and fructose), fats, and proteins can lead to hepatocyte lipid accumulation and liver steatosis [1], leading to further inflammatory hepatic environment to develop steatohepatitis [2]. Free fructose is absorbed directly from the intestinal lumen via glucose transporter 2 (GLUT2/SLC2A2) and 5 (GLUT5/SLC2A5) [3]. Once in the portal circulation, ingested fructose is targeted to liver hepatocytes via GLUT2 and GLUT8 (SLC2A8), which are glucose and fructose transporters [4,5]. Ingested fructose is almost completely extracted from the portal blood following first-pass, with only a small fraction entering systemic circulation [6]. A systemic review and meta-analysis of controlled diet-intervention trials in nondiabetic adults revealed that ingested fructose increases the availability of intrahepatic carbohydrates metabolites, leading to *de novo* lipogenesis (DNL) and presumably more harmful effects on hepatic insulin sensitivity than the isocaloric consumption of glucose, sucrose, galactose, or other carbohydrates [7].

This steatohepatitic process often involves aberrant extracellular matrix synthesis via cytokines/chemokines that influence parenchymal and nonparenchymal cells to compensate for the hepatocyte loss [8]. This aggressive fibrogenesis can result in cirrhosis and cancer [9]. Therefore, maintaining normal metabolism and/or immune-metabolic functions through well-balanced dietary habits can prevent the development of NAFLD [10]. Identifying and targeting molecules crucial to NAFLD may demonstrate clinical benefits even against hepatocellular carcinoma [11,12].

Transmembrane 4 L six family member 5 (TM4SF5) is a membrane glycoprotein with four transmembrane domains, two extracellular loops, an intracellular loop, and both cytosolic tails [13]. TM4SF5 is involved in hepatic tumorigenesis and tumor progression [14,15], and in fibrotic phenotypes in CCl_4_-administered mice [16]. Systemic TM4SF5 overexpression in mice promotes non-alcoholic steatohepatitis (NASH)-associated fibrosis via alternative signal transduction in hepatocytes [17]. TM4SF5-dependent crosstalk between hepatocytes and macrophages is also involved in NASH development following a chronic high-fat diet, a methionine-choline deficient diet, or CCl_4_-induced models [18]. However, whether TM4SF5 is involved in non-alcoholic steatosis via abnormal diets such as excessive fructose intake remains unknown. Membranous TM4SF5 forms protein–protein complexes and plays role in regulating its spatio-temporal expression and activity and those of its binding partners, leading to different intracellular signal transduction pathways [19]. TM4SF5 binds to CD151, CD133, CD44, epidermal growth factor receptor (EGFR), and integrin α5, among other partners, thereby forming massive protein–protein complexes in both plasma and endosomal membranes, called TM4SF5-enriched microdomains (T_5_ERMs) [19,20], similar to tetraspanin-enriched microdomains [21]. TM4SF5 also binds to the transporters of amino acids and other metabolic molecules, likely regulating cellular metabolic pathways [22]. A better understanding of how hepatic TM4SF5-mediated regulation of metabolic pathways and remodeling of inflammatory environment may foster the development of diagnostic and/or preventive reagents against TM4SF5-dependent NAFLD.

Our pilot experiments in which *Tm4sf5*^−/−^ knockout (KO) mice fed a high-sucrose diet showed significantly less hepatic accumulation of triacylglycerols than normal wild-type (WT) mice. Thus, we hypothesized that TM4SF5 might modulate the metabolism of fructose via protein–protein interactions for NAFLD. We found that GLUT8 plays roles in fructose uptake and metabolism in hepatocytes via changes in its intracellular locations, depending on the expression of and its binding to TM4SF5, during high-fructose diet for hepatic steatosis.

## 2 Materials and methods

### 2.1 Cells

Endogenously TM4SF5-expressing liver-cancer cell lines (Huh7 and HepG2 cells) were purchased from the Korean Cell Line Bank (Seoul, Korea). HEK293FT cells were purchased from Thermo Fisher Scientific (R7007, Waltham, MA, USA). These cells were cultured in DMEM (SH30243.01, Hyclone, Logan, UT, USA) supplemented with 10% FBS (F0600, GenDEPOT, Barker, TX, USA) and penicillin/streptomycin (CA005, GenDEPOT) at 37 °C in a 5% CO_2_ environment. All cell lines were sub-cultured every three days and routinely monitored for mycoplasma contamination.

### 2.2 *TM4SF5* suppression via lentiviral infection

HEK293FT cells at a 50–60% confluency were transfected with shTM4SF5 plasmids by polyethyleneimine (PEI, Linear, MW 25000, Polysciences Inc., Warrington, PA, USA). Short hairpin RNAs (shRNAs) targeting the human TM4SF5 sequence cloned into the lentiviral vector pLKO.1 (Addgene) are listed in Table 1.

**Table 1:** The Sequences for the shRNA targets and primers for PCR reactions.

Gene name	Target sequence	
shTM4SF5_#2_	5′-CCGGACCATGTGTACGGGAAA ATGTGCCTCGAGGCACATTTTCCCGTACACATGGTTTTTTG-3′	
shTM4SF5_#4_	5′-CCGGCCATCTCAGCTTGCAAGT CCTCGAGGACTTGCAAGCTGAGATGGTTTTTG-3′	
shTM4SF5_#12_	5′-CCGGTGGACCCAGATGCTTAATG AACTCGAGTTCATTAAGCATCTGGGTCCATTTTTG-3′	
	Primer sequence: Forward	Primer sequence: Reward
*mTm4sf5*	(#1) 5′- CTTGCTCAACCGCACTCTAT-3′ (#2) 5′-CGAATTGGACCCAAATGCCTT AAT-3′	(#1) 5′-ATCCCACACAGTACTATCTCCA-3′ (#2) 5′-CGCCTCACACAAATTCCAAAG-3′
*mKhk*	5′-GGACAGTGCAGGAGTTGGAT-3′	5′-GGACATCATCAATGTGGTGG-3′
*mSrebp1*	5′-GGAGCCATGGATTGCACATT-3′	5′-GGCCCGGGAAGTCACTGT-3′
*mChrebp*	5′-TCTGCAGATCGCGTGGAG-3′	5′-CTTGTCCCGGCATAGCAAC-3′
*mAcly*	5′-GCCAGCGGGAGCACATC-3′	5′-CTTTGCAGGTGCCACTTCATC-3′
*mAcc*	5′-ACATTCCGAGCAAGGGATAAG-3′	5′-GGGATGGCAGTAAGGTCAAA-3′
*mFasn*	5′-AGACCCGAACTCCAAGTTATTC-3′	5′-GCAGCTCCTTGTATACTTCTCC-3′
*mDgat2*	5′-GCGCTACTTCCGAGACTACTT-3′	5′-GGGCCTTATGCCAGGAAACT-3′
*mEno1*	5′-ATCTTTGACTCCCGTGGGAATC-3′	5′-AGCGGGTCTTATCATTGTCTCG-3′
*mPklr*	5′-CATCCCTGCCTTGATCATCT-3′	5′-TATCGACTCAGAGCCTGTGG-3′
*mG6pc*	5′-GACTCGCTATCTCCAAGTGAAT-3′	5′-CCAGATGGGAAAGAGGACATA G-3′
*mSlc37a4*	5′- TGGATTTCTGGCTGGCTTAC-3′	5′- CCCATCTTGGTGCGGATATT-3′

### 2.3 Animals and diets

WT, *Tm4sf5* knockout (*Tm4sf5*^−/−^ KO), and hepatocyte-specific Tm4sf5-overexpressing [via the albumin promoter conjugated to the *Tm4sf5* gene (AT, *Alb*-Tm4sf5)] transgenic C57BL/6 male mice were used for the *in vivo* experiments. Systemic KO mice were developed by targeting Exon1 (5′-CCACCTGGACGGACGGCAACCTCAGC-3′) in *Tm4sf5* [17]. Trials to generate conditional KO or liver-specific KO mice have failed. All the experimental mice were maintained under temperature-controlled (25 °C), free-moving conditions on a 14-h dark, 10-h light cycle. All the animal procedures were performed in accordance with the Seoul National University Laboratory Animal Maintenance Manual and were approved (SNU-160527-10) by the Institutional Review Board of the Institute of Laboratory Animal Resources Seoul National University (SNU-IACUC). At 6 weeks of age, C57BL/6 male mice had *ad libitum* access to tap water and either a normal chow diet (NCD), an adjusted-calorie diet (ACD) with a high sucrose diet [HSuD; 325 g/kg sucrose, Teklad custom diet, #06416, ENVIGO), or a high-fat diet (HFD, Research diets, D12492) for 10 weeks. Mice on NCD or HFD were supplied with filtered water with or without excessive fructose [30% (w/v)] with replacements every three days, which enhanced hepatosteatosis and DNL and accounts for 32% of daily caloric intake in NCD-fed C57BL/6 mice [23].

### 2.4 Body weight and food intake analyses

Age-matched animals were randomly grouped for the experimental conditions under HSuD, NCD, NCFD, HFD, or HFFD for 10 weeks. During the diet periods, the body weights of the animals were measured every week and graphed at mean ± standard deviation values. In addition, the amount of food intake by the animals (n = 5) during the HSuD was measured twice every week, leading to calculations (g/animal/day) for a graph.

### 2.5 Analysis of the acutely excessive fructose intake models

Seventeen-week-old WT or KO male mice were fed NCD or HFD for 1 week, starved for 5 h (8 am–1 pm), and then gavaged (i.e., orally injected) with either water alone or filtered water with fructose (4 g/kg). Higher dose fructose for human or mice is recently suggested to be ∼3 or >0.5 g/kg/day, respectively, leading to exposure to higher (or excessive) fructose concentrations in the liver [24]. After 90 min (fructose transit time from the mouth to the liver [25]), the animals were sacrificed and their blood and liver tissues were analyzed. Alternatively, 14-week-old WT or KO male mice (n = 5) were similarly processed before analyses of the peripheral fructose levels (see below), blood pressures (BP), or heart rates. Regarding GLUT8 suppression, the animals were intravenously injected with siRNA against control (Thermo Fisher; Cat #: 4390844) or GLUT8 (Thermo Fisher; ID: 102878) twice per week at 2.5 mg/kg. The BP-2000 Blood Pressure Analysis System (Visitech Systems, Inc., NC, USA) was used to measure blood pressures (BP_sys_ for systolic BP; BP_dia_ for diastolic BP) and the heart rates of the animals. As a control, the animals without acute fructose injection were also analyzed in parallel.

### 2.6 Analysis of blood fructose levels

Ten weeks after diet initiation, portal-vein blood was collected using a syringe fitted with a 23G needle (without plunger) after abdominal surgery to expose the vein in anesthetized (30% isoflurane in propylene glycol) mice. In addition, peripheral blood samples were collected from mice with an acute fructose intake (see above). Analyses of the fructose levels in plasma were performed using a fluorometric fructose-assay kit (ab241022; Abcam). Glucose interference was removed using the Sample Cleanup Mix, according to the manufacturer's protocols.

### 2.7 GTT and ITT analyses

Mice were fasted for 16 h (GTT) or for 6 h (ITT) with fresh water in new cages. After measurement of fasting BW and an initial blood-glucose level, either 2 g/kg d-Glucose (G8280, Sigma Aldrich) in PBS or 0.5 U/kg recombinant insulin (91077C, Sigma Aldrich) was intraperitoneally injected, before measurements of blood-glucose levels taken at 0, 30, 60, and 120 min after the injection.

### 2.8 Hepatic lipidomic analysis

Hepatic lipids were extracted as a previously described [26,27], with using freeze-dried liver tissue (10 mg) from both WT and *Tm4sf5*^−/−^ KO C57BL/6 male mice (*n* = 7 per group at 16-weeks of age) under ACD conditions with a high-sucrose content (325 g/kg) for either 3 or 10 weeks. A two-way ANOVA was performed to determine statistical significance for the multiple comparisons.

### 2.9 Hepatic lipid analysis

Assay kits for free fatty acid (ab65341, Abcam), triglycerol (ETGA-200, Bioassay Systems, Hayward, CA, USA), cholesterol, and HDL (ab65390, Abcam) were used to measure their levels from the liver tissues saved from the experimental diets

### 2.10 Western blot analysis

Cells (2–3 × 10^6^ cells/100 cm cell-culture dish) were transfected with the indicated cDNA constructs (pCMV-GLUT2-HA or pCMV10-GLUT8-FLAG_3_) using PEI (polyethyleneimine). After 24 h, the cells were starved with glucose-free DMEM media containing 5% dialyzed (3 kDa MW cut-off) FBS and 1% penicillin/streptomycin (P/S, Thermo Fisher Scientific). After 16 h, cells were treated with fructose (450 mg/dL, Sigma) prior to harvest using RIPA buffer. The primary antibodies used in the study were an antibody against KHK (sc-377411), premature-SREBP1 (sc-365513), mature-SREBP1 (sc-8984), DGAT1 (sc-271934), DGAT2 (sc-293211), β-actin (sc-47778), and GLUT2 (sc-518022) from Santa Cruz Biotechnology Inc. (Santa Cruz, CA, USA); antibodies against ACC (#3676S), pS^79^-ACC (#11818S), FASN (#3189 or #3180S), and FLAG (#2368S) from Cell Signaling Technology; an antibody against GLUT8 (ab168779) from Abcam; an antibody against FLAG (Ap1013a) from ABGENT; and a custom-designed antibody against TM4SF5 (Pro-Sci, Poway, CA, USA).

### 2.11 Immunofluorescence

Cover glasses were precoated with 10 μg/ml fibronectin (35600, BD Biosciences, San Jose, CA, USA) in PBS at 4 °C for 16 h, and then briefly washed three times with PBS. Cells on these precoated cover glasses were transfected with the indicated cDNA constructs. Following transfection for 24 h, the media was changed to starvation media [Glucose-free DMEM media containing 5% dialyzed (3 kDa cut-off) FBS and 1% P/S]. After 16 h, the cells were treated with fructose (450 mg/dL, Sigma Aldrich) for the periods indicated. The cells (on cover glasses) were then processed for immunofluorescence. Primary antibodies were diluted in 1% BSA in PBS (1:500 for anti-FLAG [NB600-344, Novus Biologicals, Littleton, CO, USA], 1:500 for anti-HA [Cell Signaling Technology], 1:400 for anti-LAMP2 [sc-18822, Santa Cruz Biotechnology]), and fluorescent secondary antibodies were incubated at 1:400 (Alexa Fluor 488 [mouse: A21202], Alexa Fluor 555 [rabbit: A31572] from Invitrogen, or DyLight 405 [goat: 705-475-147] in PBS for 1 h at room temperature (RT). Random cells were visualized using a Nikon Eclipse Ti microscope with a C2 confocal system (Nikon, Japan).

### 2.12 Immunoprecipitation

Huh7 cells were transfected with the indicated cDNA constructs using PEI and processed, as explained in immunofluorescence, before immunoprecipitation. Cells were lysed using immunoprecipitation (IP) lysis buffer (40 mM HEPES pH 7.4, 150 mM NaCl, 1 mM EDTA, and 0.5% Triton X-100) containing protease inhibitors (Thermo Fisher Scientific), before centrifugation at 12,000×*g* for 20 min at 4 °C to save the supernatants.

### 2.13 *Quantitative real-time PCR* (qRT-PCR)

PCR using total RNA from cells and from mouse-liver samples was prepared and analyzed for RT- or qRT-PCR, as explained previously [28]. The primers are listed in the Table 1.

### 2.14 Statistical analyses

For statistical analyses, all data points were tested for normality using either the D'Agostino-Pearson or Shapiro–Wilk normality test. Student's *t* test or one-way ANOVA was performed for those passing a normality test using Tukey's or Dunnett's multiple comparison post-test. Mann–Whitney or Kruskal–Wallis test was performed for those not passing a normality test. For samples involving the analysis of two factors for significance, a two-way ANOVA was performed. Prism software (version 7; GraphPad Software) was used for performing all the tests. A value of *p* < 0.05 was considered statistically significant.

## 3 Results

### 3.1 *Tm4sf5*-dependent acylglycerol accumulation in the livers of mice fed a high-sucrose diet

**Figure 1:**
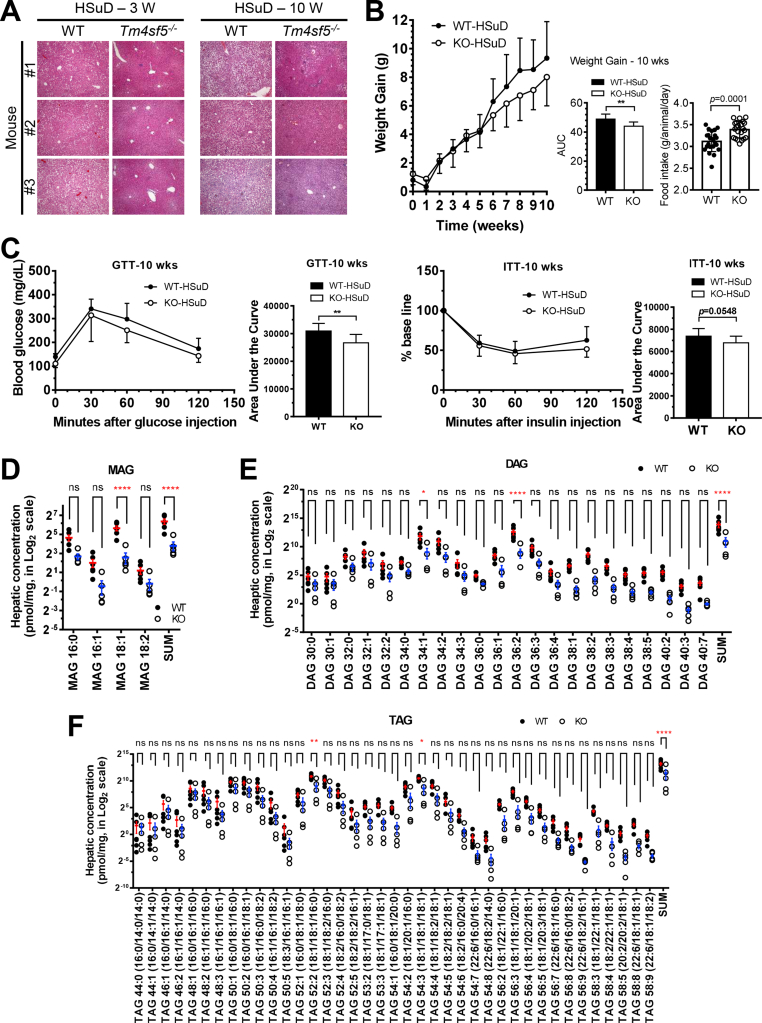
***Tm4sf5***^**−/−**^**KO mice showed lower hepatic acylglycerols levels, compared to WT mice.** Six-week-old C57BL/6 WT and *Tm4sf5*^−/−^ KO male mice (n = 10) were fed adjusted-calorie diet (ACD) with a high sucrose content diet (325 g/kg, HSuD) for either 3 or 10 weeks. (A) Randomly selected mouse-liver sections underwent H&E staining. (B) Each animal's BW was measured every week and graphed for 10-week periods, whose area under the curves (AUC) were calculated and graphed. In addition, amounts of food intake by WT and KO mice were measured and compared at mean ± standard deviation values (g/animal/day) for the diet period. (C) GTT and ITT were performed using an intraperitoneal injection of either glucose or insulin in mice fed HSuD for 10 weeks. AUC values were also calculated and graphed. (D to F) Randomly selected WT mouse livers (n = 6) and KO mouse livers (n = 5) from 10-week ACD mice were processed for LC-MS/MS-based lipidomic analyses. Among the lipids analyzed, MAG, DAG, and TAG levels were graphed. ∗, ∗∗, ∗∗∗, and ∗∗∗∗ depict *p* values less than 0.05, 0.01, 0.005, or 0.001to represent statistical significance using student *t* test (B and C) or a two-way ANOVA (D to F).

We focused on the roles of TM4SF5 in non-alcoholic fatty liver and/or abdominal obesity, after observations on abdominal obesity and NASH in the *TM4SF5*-genetic animal models [17]. As a pilot experiment, we observed a difference in fat accumulation between normal WT and *Tm4sf5*^−/−^ KO mice (n = 10) fed a calorie-adjusted purified low-fat and high-sucrose content diet (HSuD; sucrose at 325 g/kg, Teklad # 06416) for either 3 or 10 weeks. This adjusted-calorie diet (ACD) was purchased from Harlan Laboratories (www.harlan.com) and included carbohydrates (64.7% by weight and 69.8% kcal), proteins (18.6% by weight and 20.1% kcal), and fats (4.2% by weight and 10.2% kcal). Because of higher sucrose content, we speculated that TM4SF5 might be involved in sucrose or fructose/glucose metabolism. Three or 10 weeks of ACD consumption by normal WT mice showed that they accumulated more liver fats and lipids than*Tm4sf5*^−/−^ KO mice, indicating that WT mice had ACD-induced fatty livers (Figure 1A). Furthermore, the alanine transaminase (ALT), aspartate transaminase, total cholesterol, and triglycerol (TG) blood-sample levels in mice following 10 weeks of this diet were not significantly different between the WT and KO groups, although the ALT levels were insignificantly lower in KO mice than in WT mice (Fig. S1A). The KO mice showed significantly less gains of body weights (BWs) than the WT mice, although the KO mice had ingested more food (Figure 1B), suggesting that TM4SF5 might regulate dietary carbohydrate metabolism. In addition, glucose and insulin tolerance tests (GTT and ITT, respectively) via intraperitoneal injections of glucose or insulin showed no significant differences in these tolerances between the 3-week groups of WT and KO mice (Fig. S1B). However, in the 10-week diet groups, KO mice showed more tolerance (insulin-sensitive blood glucose levels) using both tests (Fig. 1C). To better understand the hepatic lipid-metabolism changes caused by the *Tm4sf5* knockout, we performed lipidomic analyses of lyophilized liver tissue from seven randomly chosen mice from each group. Among the many different fats and lipids analyzed, the monoacylglycerol, diacylglycerol, and triacylglycerol (MAG, DAG, and TAG, respectively) levels using specific carbon lengths (i.e., MAG: 18:1; DAG: 34:1; DAG: 36:2; TAG: 52:2; TAG: 54:3) were significantly lower in KO mice than in WT mice (Figure 1D,E, and F). These observations suggest that ACDs with higher carbohydrate or sucrose (as a disaccharide containing fructose) content may lead to TM4SF5-dependent NAFLD with acylglycerol accumulation.

### 3.2 A high-fructose diet (NCFD) causes hepatic TAG accumulation and lipogenesis in mice in a TM4SF5-expression dependent manner

**Figure 2:**
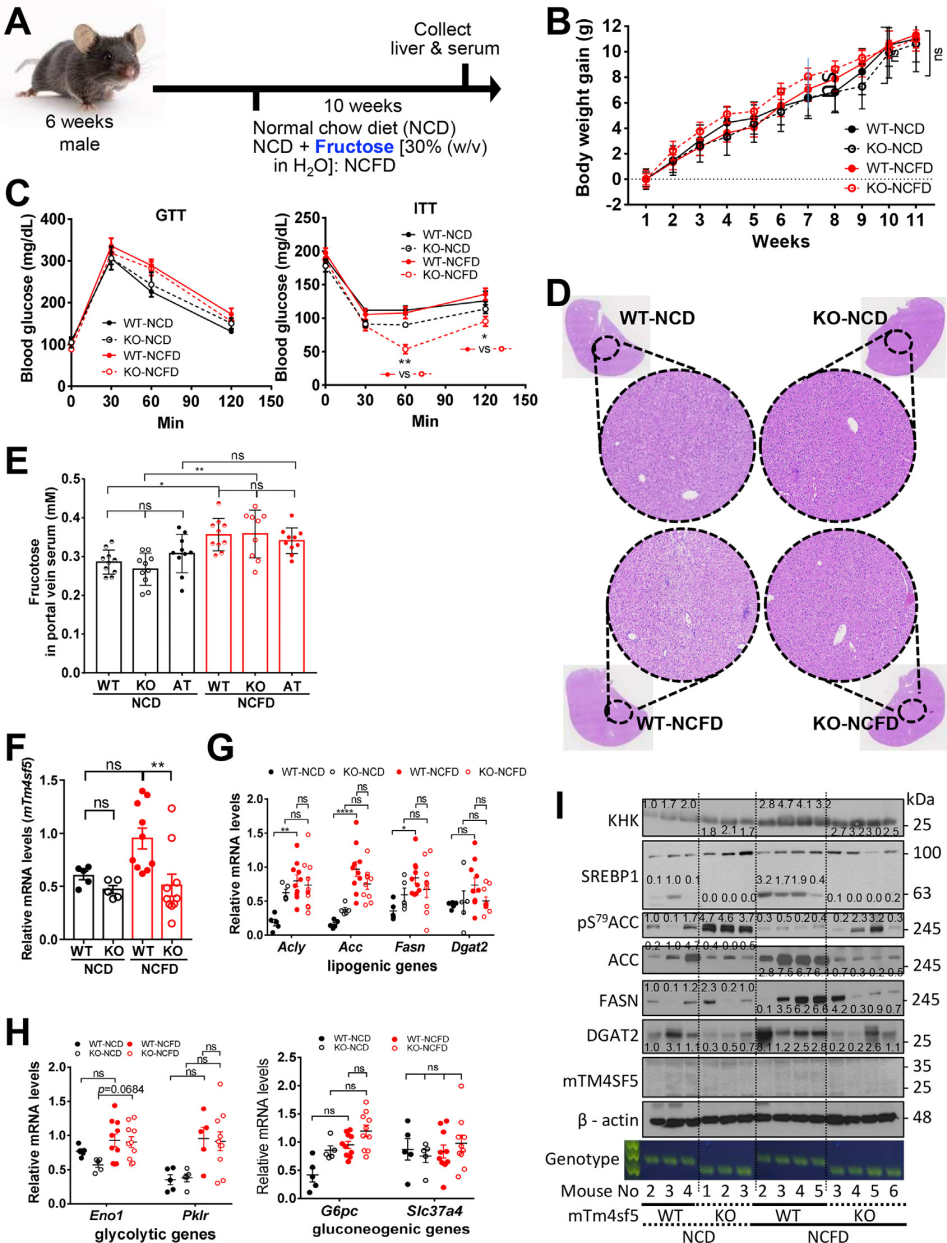
**Lipogenesis in WT, but not in *Tm4sf5***^**−/−**^**KO mice, was promoted following an excessive fructose supply**. Six-week-old C56BL/6 WT and *Tm4sf5*^−/−^ KO male mice were fed *ad libitum* with either H_2_O (n = 5) or fructose 30% w/v in H_2_O (NCFD, n = 10) in addition to a normal chow diet (NCD) for 10 weeks. At the end of this period, the livers were collected and analyzed. (A) Diet schedule. (B) Each animal's BW was measured every week and the changes graphed. A two-way ANOVA showed no significance (ns) between groups. (C) Eight weeks into their NCD or NCFD period, the mice were fasted for 16 h (GTT) or for 6 h (ITT) with fresh water in new cages, before measuring their initial BWs. GTT and ITT were performed using intraperitoneal injections of either 2 g/kg d-glucose in PBS or 0.5 U/kg recombinant insulin. Blood-glucose levels were determined 30, 60, and 120 min after the injections. The *p* values were calculated using a one-way repeated-measure ANOVA and Dunn's test. ∗*p* < 0.05 and ∗∗*p* < 0.01 were considered significant. (D) Liver-tissue samples from experimental mouse groups were stained using H&E, and representative (random) analysis-images are shown. (E) Portal-vein blood samples [WT, KO, and *Alb*-Tm4sf5 TG (AT) mice, n = 10] from mice fed NCD or NCFD for 10 weeks were collected for serum analysis of fructose levels. Outliers (at most, one in each group) were excluded. *P* < 0.05 values indicate statistical significance (∗ for *p* < 0.05, ∗∗ for *p* < 0.01 and ∗∗∗ for *p <* 0.005) using a one-way ANOVA. ns = not significant. (F to H) Liver tissue was analyzed using qRT-PCR for mouse *Tm4sf5* (*mTm4sf5*, F) or other indicated mRNAs (G and H) or using immunoblots (I) for protein levels of the indicated molecules for WT and KO mice fed NCD or NCFD. ∗, ∗∗, ∗∗∗, and ∗∗∗∗ indicate statistical significance (*p* < 0.05, 0.01, 0.005, and 0.001, respectively) using a one-way ANOVA. ns = not significant. The data shown represent three independent experiments.

Because hepatic acylglycerol accumulation in a TM4SF5-dependent manner is caused by a diet with higher sucrose that is broken down into fructose, which is a major constituent of various dietary and drinks, we speculated whether excessive fructose consumption could lead to NAFLD [29]. Thus, we investigated whether excessive fructose intake caused fatty-liver phenotypes with hepatic TAG accumulation in a TM4SF5-dependent manner. Six-week-old male C57BL/6 WT and *Tm4sf5*^−/−^ KO mice were fed a normal-chow diet (NCD) and were given *ad libitum* access to either plain drinking water (i.e., NCD) or water containing 30% (w/v) fructose (i.e., NCFD) for ten weeks (Figure 2A). During these ten weeks, the BWs of all the mice increased similarly, with no significant difference in BW gains among the animal groups (Figure 2B). The 10-week WT mice group fed NCFD, but not the KO mice group, showed reduced blood glucose levels (∗, *p <* 0.05), whereas both groups showed decreased blood disaccharide to contain TAG levels (Fig. S2A, upper). However, the hepatic triglyceride (TAG) levels under NCFD conditions were not significantly increased in WT mice, and KO mice under NCFD maintained the levels similarly to the basal level under NCD-only conditions (Fig. S2A, bottom and left). The NCD-based basal liver levels of high-density lipoprotein (HDL) cholesterol were significantly higher in KO mice than in WT mice. However, with the NCFD, hepatic HDL levels in KO mice were not significantly higher than those in WT mice (Fig. S2A, bottom and right). In addition, total free fatty acid (FFA) and cholesterol levels in liver were not different among the groups, and the NCFD in both WT and KO mice showed no significant decrease in the total hepatic cholesterol levels (Fig. S2B). These results suggest that TM4SF5 expression may be involved in NCFD-mediated lipid accumulation (i.e., fatty liver disease likely caused via increased TAG and decreased HDL). We next assessed insulin significance during possible TM4SF5-dependent steatosis via NCFD. Glucose-tolerance testing (i.p., glucose, 2 g/kg) resulted in both WT and KO mice showing glucose intolerance following NCFD (Figure 2C, left). Insulin-tolerance testing (i.p., recombinant insulin, 0.5 U/kg) of KO mice showed significant insulin sensitivity following NCFD, but WT mice did not (Figure 2C, right).

Liver hematoxylin and eosin (H&E) staining revealed steatotic phenotypes in WT mice, but not in KO mice, under NCFD conditions (Figure 2D). The fructose levels in the portal veins (gut to liver) of animals increased independently of TM4SF5 expression under NCFD conditions; WT mice, TM4SF5-overexpressing transgenic mice (under albumin-promoter control for hepatocyte expression; *Alb*-*Tm4sf5* transgenic mice, AT), and KO mice all showed similarly enhanced fructose levels in their portal veins (Figure 2E). The fructose levels in the peripheral blood circulation measured 1.5 h after (acutely) oral injection of excessive fructose (4 g/kg in H_2_O) were independent of TM4SF5 expression (Fig. S2C). We also found that the blood pressures and heart rates in KO mice showed trends lower than those of WT mice, independent of the oral injection of excessive fructose (Fig. S2D), which may be an interesting issue to study in future.

NCFD conditions did not significantly increase the *mTm4sf5* mRNA levels in WT mice to a level significantly different from those in KO mice (Figure 2F, left). Under NCFD conditions, ketohexokinase (*Khk*) mRNA, coding for the first enzyme involved in intracellular fructose metabolism, was also increased in WTs but not in KO mice (Fig. S2E, left). In addition, the level of sterol regulatory element-binding protein 1 (*Srebp1*) mRNA showed a decrease under NCFD conditions in KO mice, compared with that in WT mice, and carbohydrate response element binding protein (*Chrebp*) mRNA was not significantly changed either by *Tm4sf5* expression or NCFD conditions (Fig. S2E). Furthermore, WT mice, but not KO mice, fed NCFD significantly induced lipogenic genes, such as ATP citrate lyase (*Acly*), acetyl-CoA carboxylase (*Acc*), and fatty acid synthase (*Fasn*), but not diacylglycerol O-acyltransferase 2 (*Dgat2*) (Figure 2G). In addition, both glycolytic α-enolase (*Eno1*) and pyruvate kinase (*Pklr*) were not significantly enhanced in both WT and KO mice fed NCFD (Figure 2H, left), whereas the gluconeogenic glucose-6-phosphatase catalytic subunit (*G6pc*) in WT or KO mice was not significantly increased, and the glucose-6-phosphate exchanger (*Slc37a4*) in both WT and KO mice was not changed (Figure 2H, right). Furthermore, liver-tissue immunoblots from these groups showed that WT mice fed NCFD exhibited increased levels of KHK, FASN, and diacylglycerol *O*-acyltransferase 2 (DGAT2), indicating excessive fructose-mediated lipogenesis and TAG accumulation (Figure 2I). However, KO mice also showed increased levels of KHK, but not those of FASN and DGAT2, indicating that NCFD conditions led to increased fructose uptake in KO mice similar to that observed in WT mice, but not leading to lipogenesis and TAG accumulation (Figure 2I). Furthermore, WT mice showed less pS^79^ACC and more ACC expression under NCFD conditions, presumably reflecting a more energy-efficient status, whereas KO mice showed increased pS^79^-ACC levels under NCFD conditions to levels less than those of KO mice under NCD conditions (Figure 2I). Therefore, KO mice metabolize fructose without leading to lipid and TAG accumulations in the liver, unlike WT mice.

### 3.3 TM4SF5 deficiency impairs fructose-induced TAG accumulation in the liver even under high-fat diet (HFD) conditions

**Figure 3:**
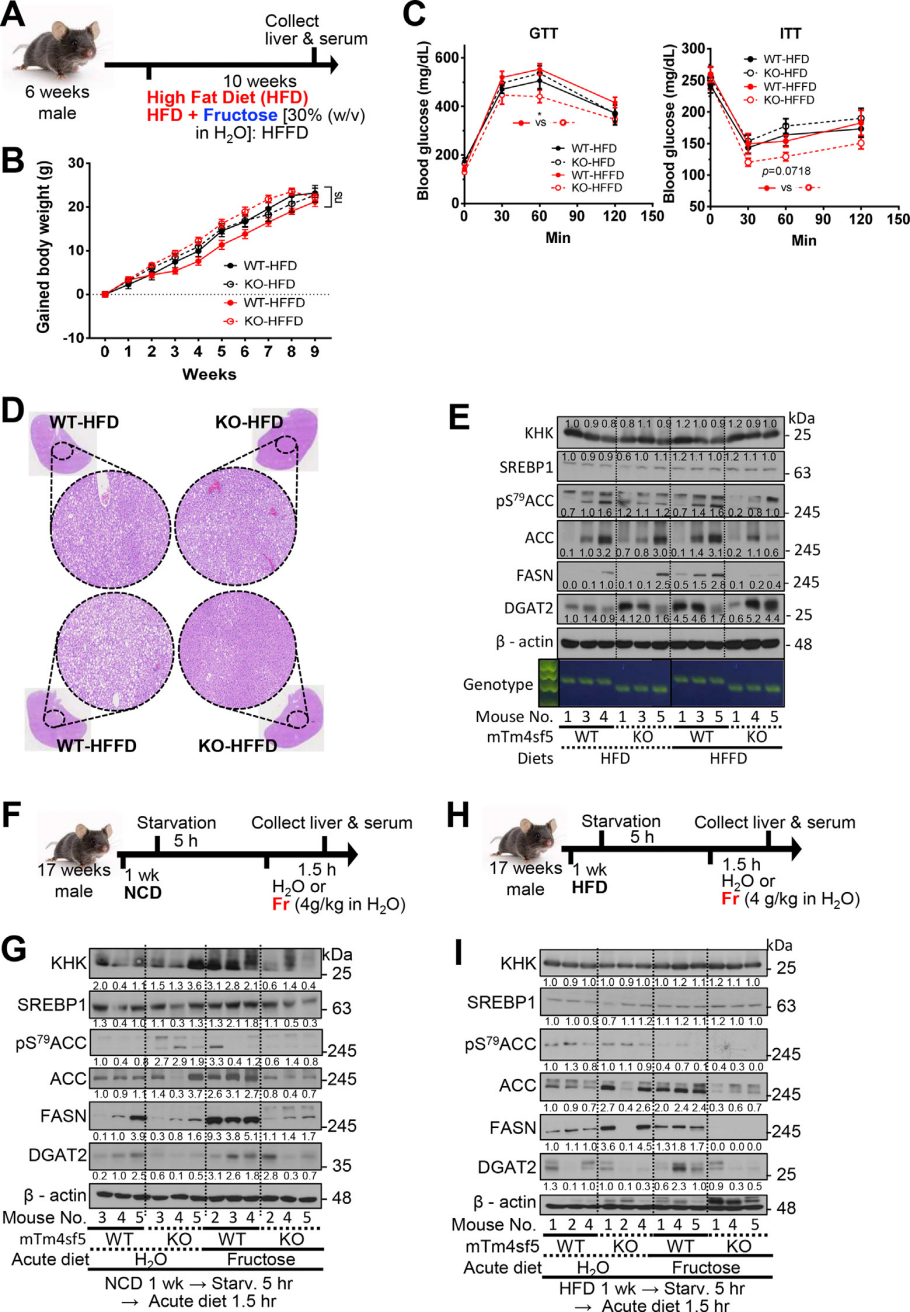
***Tm4sf5***^**−/−**^**KO mice showed less lipogenesis even under high-fructoseand fatdiet conditions.** Six-week-old C56BL/6 WT and *Tm4sf5*^−/−^ KO male mice were fed *ad libitum* either high-fat diet alone (HFD, 60% kcal fat, n = 5) or fructose 30% w/v in H_2_O in addition to HFD (HFFD, n = 5) for 10 weeks. At the end of that period, their livers were collected and analyzed. (A) Diet schedule. (B) Each animal's BW was measured every week and the changes were graphed. A two-way ANOVA revealed no significance (ns) between groups. (C) As explained in Figure 2C, after 8 weeks into their HFD or HFFD period, mice were fasted for 16 h (GTT) or for 6 h (ITT) with fresh water in new cages, before measuring their initial BWs. GTT and ITT analysis. The *p* values were calculated using one-way repeated-measure ANOVA and Dunn's test. ∗*p* < 0.05 was considered significant. (D) Liver tissue samples from experimental mouse groups were stained using H&E, and representative (random) analysis-images are shown. (E) Liver tissue was analyzed using RT-PCR (for genotyping) and using immunoblots for protein levels of the indicated molecules in WT and KO mice under HFD or HFFD. (F to I) Seventeen-week-old C56BL/6 WT and *Tm4sf5*^−/−^ KO male mice [n = 5 for the H_2_O group and n = 5 for the excessive fructose (acute fructose diet) group] were fed *ad libitum* NCD (F and G) or HFD (H and I) for one week prior to being starved for 5 h and then feeding with either plain H_2_O or via an oral injection of fructose (fructose 4 g/kg in H_2_O). After 1.5 h, their livers were collected and analyzed. (F and H) Diet schedules. (G and I) Immunoblotting using liver-tissue extracts was performed for the indicated molecules. The data shown represent three independent experiments.

The blood TAG levels were significantly reduced in both WT and KO groups under NCFD conditions for 10 weeks, whereas the hepatic TAG levels were not significantly increased in WT mice, unlike in KO mice (Fig. S2A). Therefore, we speculated that whether 10-week-fructose (30% w/v) in addition to HFD (i.e., HFFD) caused steatosis and/or steatohepatitis in the animal livers (Figure 3A). Interestingly, under HFD or HFFD conditions for 10 weeks, both WT and KO mice showed no significant difference in BW gains (Figure 3B). The blood glucose levels were not significantly different between any mouse groups (Fig. S3A, top left). The blood and hepatic TAG levels were not different between WT and KO mice under HFD conditions, whereas HFFD conditions significantly reduced the blood and hepatic TAG levels in KO mice, but not in WT mice, compared with those under HFD conditions (Fig. S3A). In addition, hepatic HDL was increased in KO mice under HFFD conditions but was decreased in WT mice (Fig. S3A, bottom and right). The total hepatic FFA levels in WT mice under HFD conditions were significantly higher than those in KO mice, whereas the hepatic FFA levels were not different between WT and KO mice under HFFD conditions. Thus, HFFD-mediated increases in the hepatic FFA levels were significantly greater in KO mice than in WT mice (Fig. S3B, left). Furthermore, the hepatic cholesterol levels among all the mouse groups were not different (Fig. S3B, right). Thus, under HFFD conditions, the livers of WT mice accumulated TAG while those of KO mice accumulated FFA (although at levels similar to those of WT mice), indicating that unlike WT mice, KO mice could not accumulate TAG due to the esterification of FFAs metabolized under HFFD conditions. Under HFFD conditions, GTT analysis of WT mice again showed glucose intolerance, but KO mice were glucose tolerant (Figure 3C, left) and the ITT of KO mice showed significant insulin sensitivity under HFFD conditions, but not that of WT mice (Figure 3C, right).

Furthermore, under HFD or HFFD conditions, liver H&E staining showed more aggravated steatosis in WT mice than in KO mice; WT mice showed more lipid accumulation following HFFD, but KO mice showed a reduction (Figure 3D). Thus, KO mice actively cleared fats and lipids rather than accumulated them in the liver. Liver immunoblots showed that the ketohexokinase (KHK) levels were not different among the mouse groups, indicating that HFFD conditions may not cause changes in KHK expression, presumably because of the marked effects on the expression following HFD alone. However, the pS^79^ACC levels were comparably maintained following HFFD in WT mice but not in KO mice (i.e., the levels were only slightly decreased), whereas the FASN levels were increased following HFFD in WT mice, but not in KO mice (Figure 3E). In addition, the HFFD conditions increased Diacylglycerol O-acyltransferase 2 (DGAT2) expression in WT mice to the levels observed in KO mice under HFD conditions even without excessive fructose intake (Figure 3E). Thus, KO mice efficiently metabolized fructose, leading to insufficient FFAs required for TAG accumulation in the liver and more HDL.

Because the findings regarding how 10 weeks of HFD conditioning might affect TM4SF5-dependent mouse steatosis were not easily interpreted, we adopted a short-term approach to assess the effects of excessive fructose intake with NCD- or HFD-pretreatment for one week. Seventeen-week-old male C57BL/6 mice fed NCD for 1 week were starved for 5 h and then were orally injected with vehicle alone (H_2_O) or fructose (4 g/kg in H_2_O), 1.5 h before collection of their livers and sera for analysis (Figure 3F). Using this short-term approach, fructose access in WT mice, but not in KO mice, clearly increased the KHK levels (presumably to support the increased fructose uptake), leading to increased ACC, FASN, and DGAT2 expression (Figure 3G). In parallel experiments, other 17-week-old male C57BL/6 mice were fed a HFD for one week and then were starved for 5 h before an oral injection of vehicle alone (H_2_O) or fructose (4 g/kg in H_2_O), 1.5 h before collection of their livers and sera for analysis (Figure 3H). In these mice, the KHK levels were not different between groups, and the WT and KO mice with fructose access showed lower pS^79^ACC levels. In addition, WT mice showed increased ACC, FASN and DGAT2 levels after fructose intake, but KO mice showed decreased levels lower than those observed in WT mice (Figure 3I). Additionally, such TM4SF5-mediated effects following an acute intake of excessive fructose were not observed when the mice were not acutely injected with excessive fructose (following NCD or HFD for 1 week) as a negative control (Fig. S4). These observations indicate that fructose intake into WT mice led to lipogenesis, whereas the *Tm4sf5* deficiency led to less fructose uptake (in the case of NCFD) and less lipogenesis even following excessive fructose intake.

### 3.4 Fructose uptake and crosstalk between TM4SF5 and GLUT8 intracellular localization

**Figure 4:**
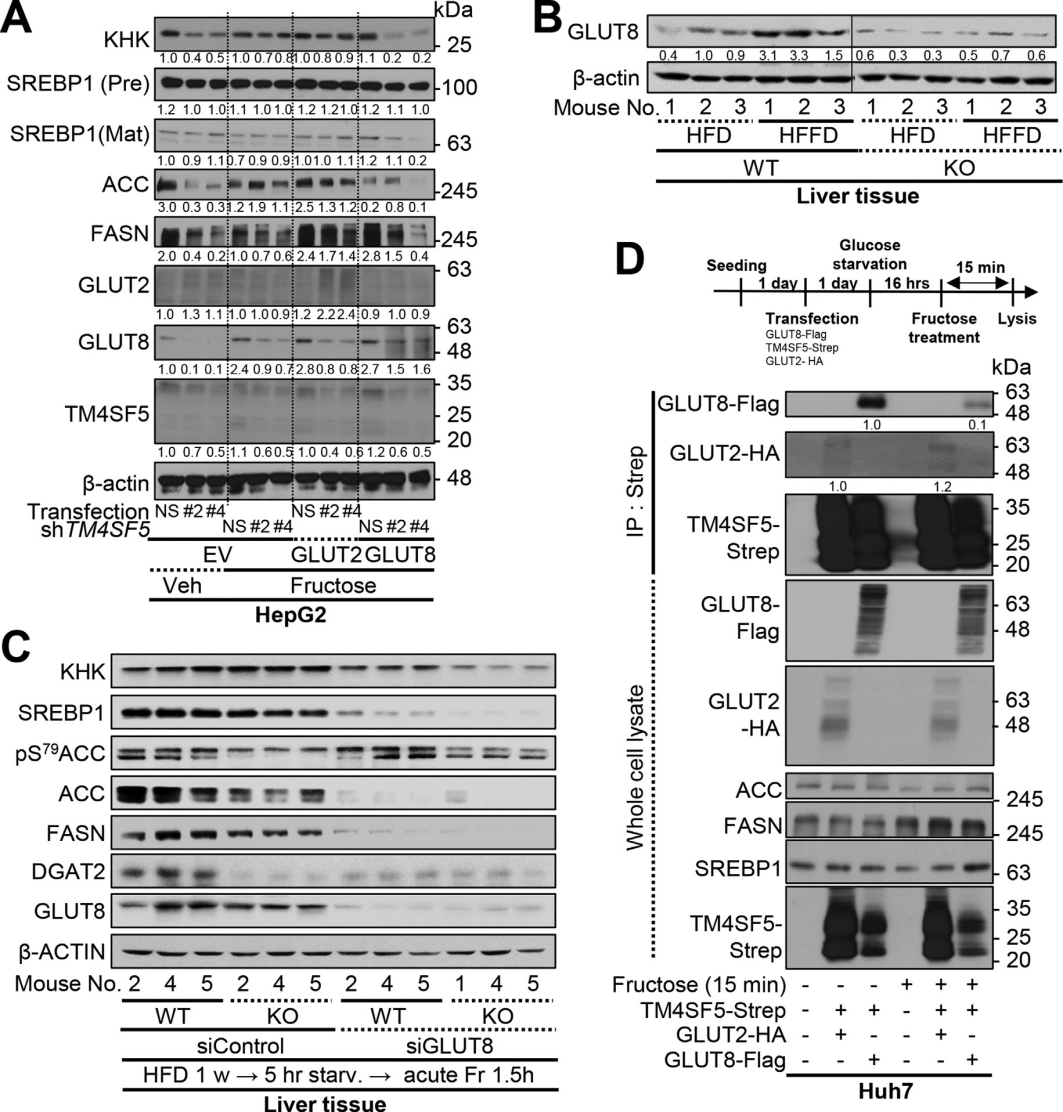
**TM4SF5-mediated lipogenic activity upon excessive fructose intake involves GLUT8.** (A) HepG2 cells (endogenously expressing TM4SF5) were transiently transduced with lentivirus for either a non-specific sequence (NS) or *TM4SF5* target sequences (#2 or #4) together with transfections of either empty vector (EV-FLAG), or GLUT2-HA and GLUT8-FLAG plasmids for 24 h, glucose-starved for 16 h, and then treated with fructose (450 mg/dL) for 30 min prior to whole cell lysate preparation for standard western blot analysis. (B) Tissue extracts from livers of WT or KO mice fed HFD or HFFD for 10 weeks were prepared for immunoblotting. (C) WT or KO mice were fed with HFD for 1 week and siRNA against control or GLUT8 sequence (siGLUT8) was intravenously injected twice per week (2.5 mg/kg/animal). The animals were starved for 5 h and orally injected with excessive fructose (4 g/kg in H_2_O), 90 min before preparation of liver tissue extracts and immunoblotting. (D) Huh7 cells (endogenously expressing TM4SF5) were transiently transfected with either TM4SF5-Strep, GLUT2-HA, or GLUT8-FLAG constructs. One day later, the cells were glucose-starved for 16 h before treatment with fructose for 15 min. The cells were then harvested both for co-pulldown experiments using streptavidin-conjugated agarose beads (B) and for standard western blot analysis of the indicated molecules (C). The data shown are representative of three independent experiments.

Next, we explored how hepatocyte TM4SF5 expression might regulate fructose or glucose uptake in the context of insulin resistance. First, we transiently transfected HepG2 cells with either GLUT2 or GLUT8 (as glucose/fructose transporters) together with either non-specific siRNA (NS) or siTM4SF5 (against sequence #2 or #4) for 48 h. Immunoblots using whole cell lysates showed decreased KHK expression caused by TM4SF5 suppression when these cells expressed endogenous GLUTs in the absence of fructose treatment and exogenously overexpressed GLUT8 (but not GLUT2) in the presence of fructose treatment (Figure 4A). Furthermore, TM4SF5 suppression reduced the SREBP1, ACC, and FASN levels in these cells with or without GLUT8 overexpression and fructose treatment (Figures 4A and S5A). In addition, KO mice fed HFD or HFFD showed lower GLUT8 expression levels than WT mice (Figure 4B). Furthermore, the TM4SF5-dependent effects on increases in liver lipogenic enzymes following acutely excessive fructose intake (Figure 3I) appeared to be abolished by GLUT8 suppression (Figure 4C). Thus, without GLUT8 to transport fructose into hepatocytes, KHK, ACC, and lipogenic enzymes appeared less functionally expressed (Figure 4C). These observations suggest that TM4SF5-dependent steatotic phenotypes following excessive fructose intake may involve GLUT8.

**Figure 5:**
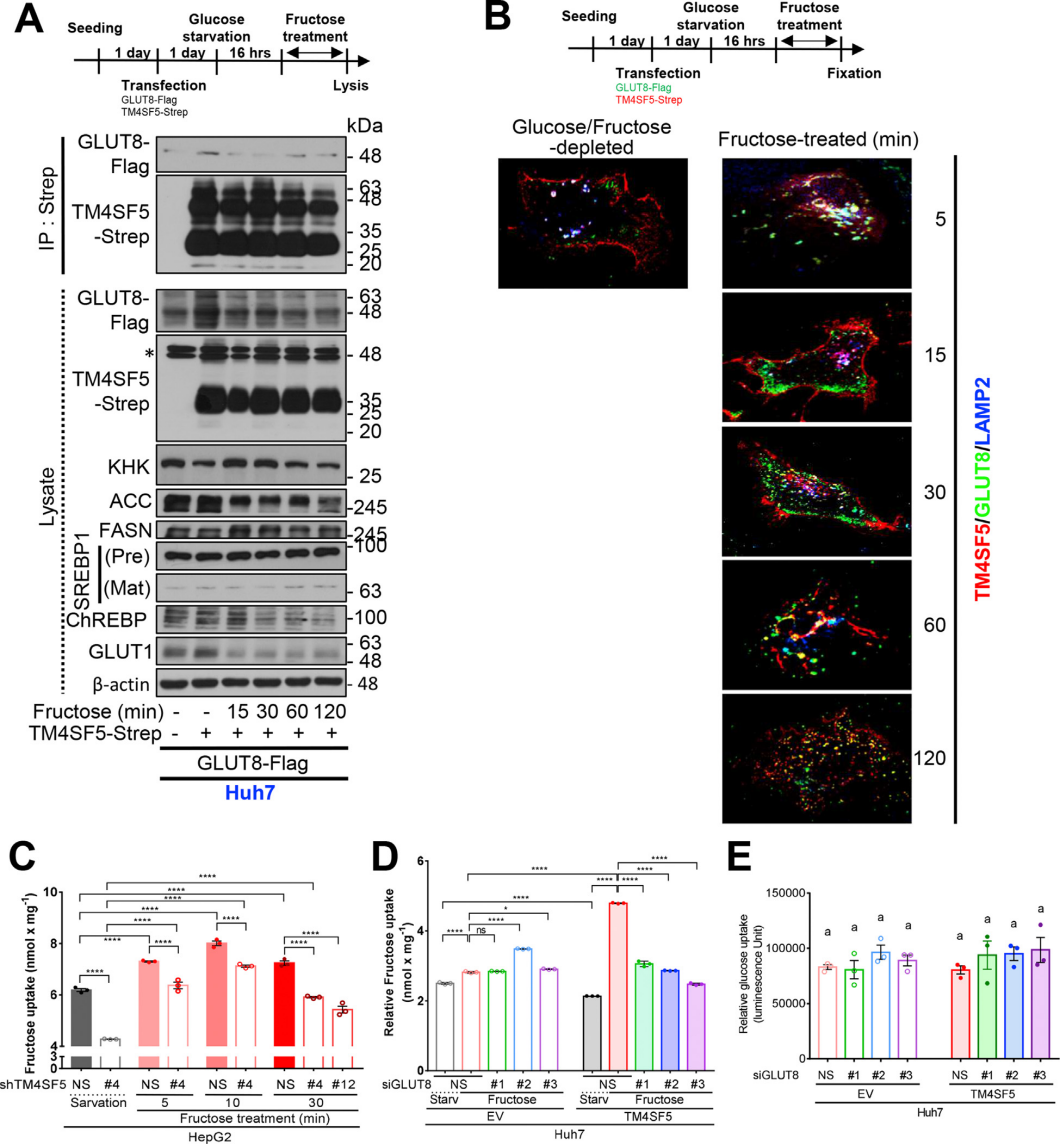
**TM4SF5 mediated intracellular translocation and activation of GLUT8 for fructose uptake.** (A and B) TM4SF5-expressing Huh7 cells were transiently transfected with TM4SF5-Strep and/or GLUT8-FLAG constructs. One day later, the cells were glucose-starved for 16 h, before being treated with fructose for various periods (0–120 min). The cells were then harvested for co-pulldown experiments using streptavidin-conjugated agarose beads, for standard western blot analysis of the indicated molecules (A) or fixed for indirect immunofluorescence microscopy (red for TM4SF5-Strep and green for GLUT8-FLAG). (C to E) HepG2 cells (C) and Huh7 cells (D and E) were transiently transfected with shRNA for either control (NS), *TM4SF5* (#4 or #12), or *GLUT8* sequence (#1, #2, or #3) together with empty vector (EV) or a TM4SF5-expression vector for 48 h. The cells were then glucose-starved for 16 h and treated with 450 mg/dL fructose for the indicated periods (min) prior to being assessed for fructose (C and D) or glucose (E) uptake. Statistical significance (∗*p* < 0.05, ∗∗*p* < 0.01, ∗∗∗*p* < 0.005, and ∗∗∗∗*p* < 0.001) was determined using one-way ANOVA and Tukey's test. ns = not significant. The data shown represent three independent experiments.

We investigated the possible linkage between TM4SF5 and GLUT8 for hepatic fructose uptake using coimmunoprecipitations. When glucose-depleted Huh7 cells were treated with fructose (15 min), exogenous GLUT8 binding to TM4SF5 was diminished, whereas the binding of exogenous GLUT2 to TM4SF5 was constitutively maintained (Figure 4D). Furthermore, when GLUT8-TM4SF5 binding was reduced, the expression levels of SREBP1 and FASN were increased and maintained compared with their levels without fructose treatment where basal GLUT8-TM4SF5 binding was greater (Figure 4D, lanes 3 and 6). After fructose treatment, GLUT8-TM4SF5 binding was transiently lost (∼15–30 min) and then recovered 60–120 min after treatment, with concomitant KHK and FASN expression levels increasing and then decreasing (Figure 5A). In addition, the SREBP1, ChREBP, and GLUT1 expression levels were not correlated with the binding patterns or KHK levels, although the expression levels of both ChREBP and GLUT1 were decreased following fructose treatment (Figure 5A). Immunofluorescence analysis of glucose-starved cells revealed more TM4SF5 in the PM and less in lysosome-associated membrane protein 2 (LAMP2)-positive lysosomes along with GLUT8 (Figure 5B, left image). With fructose treatment, the colocalization of TM4SF5 with GLUT8 transiently changed, markedly increasing within 5 min of treatment, declining between 15 and 30 min (with TM4SF5 translocating to the PM and GLUT8 to the peri-PM), and then recovering again at lysosomes by 60–120 min, as shown by co-immunoprecipitation (Figure 5A,B). Our live imaging trials using TM4SF5-mCherry and GLUT8-GFP for periods less than 15 min following fructose treatment failed to show more GLUT8 in the PM, but its localization to the PM was observed 15–30 min after fructose treatment and might have been affected by TM4SF5 (Figure 5B).

Consistently, TM4SF5 suppression resulted in significantly lower fructose uptake in glucose-starved HepG2 cells before and after fructose treatment, but both TM4SF5-positive and suppressed conditions resulted in increased fructose uptake (peaking at 10 min), which then declined afterwards (Figure 5C). GLUT8 suppression in TM4SF5-overexpressing cells, but not in endogenously TM4SF5-expressing Huh7 cells, significantly decreased fructose uptake, and GLUT8 suppression did not affect glucose uptake following fructose treatment (Figure 5D,E).

### 3.5 TM4SF5 subdomains are important for GLUT8 binding

Next, we investigated which regions of TM4SF5 bound to GLUT8. First, we performed co-precipitation of GLUT8-FLAG with TM4SF5 WT or mutant forms of TM4SF5-Strep [a glycosylation-deficient N138A/N155Q mutant (Gly^−^), a palmitoylation-deficient C2/6/9/74/75/79/80/84/189A mutant (Pal^−^, [30]), a C-terminal half-deleted mutant (TM4SF5_1-90_ with amino acids 1–90), and an N-terminal half-deleted mutant (TM4SF5_91-197_ with amino acids 91–197)]. Only the TM4SF5_1-90_ deletion mutant lost binding capacity, whereas TM4SF5_91-197_ bound to GLUT8 (Fig. S5B). Next, various point mutations in the long extracellular loop of TM4SF5 were pre-screened for binding to GLUT8, and the A132V, N138A, and V156A mutants were selected for analysis. In addition to the A132V mutant, the N138A mutant (deficient for N-glycosylation) did not lose the ability to bind to GLUT8, unlike the V156A mutant (Fig. S5C). In addition, the T157A mutant, but not the W154A and N155Q mutants, lost the ability to bind to GLUT8 (Fig. S5D). Furthermore, the N138A and W154A mutants, but not N155Q, showed significant N-glycosylation losses (at ∼35 kDa), although all three bound to GLUT8 (Fig. S4), indicating no need of TM4SF5 N-glycosylation for GLUT8 binding. Instead, the significance of the V156 and T157 residues in GLUT8 binding is likely to identify the 4th transmembrane domain of TM4SF5 for successful binding.

## 4 Discussion

**Figure 6:**
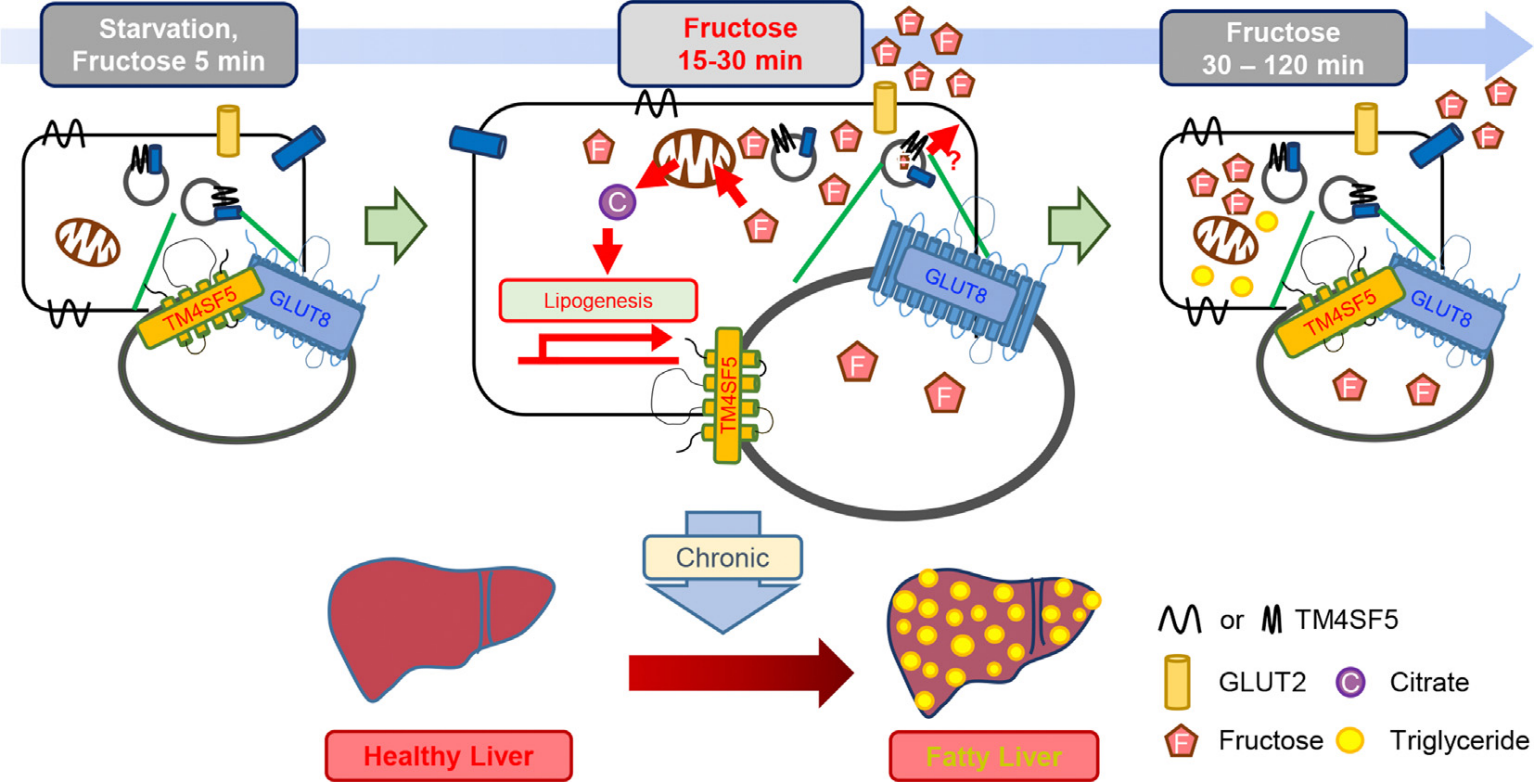
**A working model to explain the crosstalk between hepatic TM4SF5 and GLUT8 that promotes fructose uptake and leads to steatosis under high-fructose conditions.** TM4SF5-expressing hepatocytes can dynamically regulate fructose uptake, which can then be metabolized into mono-, di-, or tri-acylglycerol and lead to fructose-mediated hepatic steatosis. Upon glucose-starvation, or immediately after fructose treatment (5 min), TM4SF5 and GLUT8 were mostly colocalized in LAMP2-positive lysosomes. At later time points (15–30 min), during enhanced fructose uptake into hepatocytes, TM4SF5 and GLUT8 were translocated to PMs and separated from each other. Transported fructose could induce *de novo* lipogenesis via increased fatty acid and acylglycerol syntheses. Such chronically increased synthesis and accumulation of acylglycerols and LDLs could result in hepatic steatosis. During later periods after fructose treatment (more than 30 min), TM4SF5 and GLUT8 were translocated back to lysosomes and accompanied by a reduced capacity for fructose uptake. Under these conditions, the binding of GLUT2 to TM4SF5 was apparently unchanged by fructose treatment, and glucose uptake was irrelevant to the collaboration between TM4SF5 and GLUT8.

Here, we provide evidence that hepatocyte TM4SF5 expression can regulate both the intracellular localization and fructose-transporting activity of GLUT8 (a glucose/fructose transporter) under HFD or HFFD conditions. These TM4SF5-mediated effects involved DNL through hepatocyte accumulation of lipids, including acylglycerols and cholesterols, for hepatic steatosis. The TM4SF5 knockout or suppression caused less acylglycerol synthesis and more HDL synthesis and insulin sensitivity, leading to less steatotic phenotypes. For hepatocyte responses to either high-extracellular or high-dietary fructose, TM4SF5 affected the intracellular translocalization of GLUT8 from lysosomal membranes to PMs, causing fructose to be taken up in intracellular regions and metabolized into fatty acids and lipids. TM4SF5 constitutively bound GLUT2 but dynamically bound GLUT8 after fructose treatment; early after treatment (e.g., 5 min), TM4SF5 bound GLUT8 mostly in LAMP2-positive lysosomes, but later (e.g., ∼15–30 min) TM4SF5 and GLUT8 separated from each other during translocation from lysosomes to PMs. Although TM4SF5 and GLUT8 were localized exclusively at PMs after fructose treatment (e.g., ∼30 min), the fructose transport/metabolism greatly increased through the induction of enzyme KHK and FASN in a TM4SF5-dependent manner. At later times after fructose treatment (e.g., ∼60–120 min), TM4SF5 binding to GLUT8 was recovered at lysosomes with a coincident decline in fructose uptake (Figure 6). The intracellular movement of GLUT8 and its protein–protein interactions therefore depend on TM4SF5 expression in hepatocytes and are clearly important for fructose uptake/metabolism for excessive fructose-mediated steatosis.

TM4SF5 shares similarities with tetraspanins [31], by having four transmembrane domains with N-glycosylation and palmitoylation modifications [30]. It can form protein–protein complexes on cellular membranes, called tetraspanin-enriched microdomains (TERMs; T_5_ERMs for TM4SF5-enriched microdomains) [19]. TM4SF5 plays regulatory roles in the intracellular trafficking of other membrane proteins and receptors [32]; it binds to different membrane proteins and receptors, changing their intracellular locations between lysosomal and PMs, presumably leading to changes in signaling activity [20,22]. TM4SF5 binds to EGFR [30], integrin α5 [30,33], CD133 [34], CD151 [35], CD44 [36], CD98hc/SLC7A11 [37], SLC27A2/SLC27A5 [38], and mTOR [28]. Similarly, TM4SF5 may cause GLUT8 to be trafficked to the PM from lysosomes, where TM4SF5 and GLUT8 are localized separately presumably to transport extracellular fructose. Similarly, TM4SF5 binding to SLC27A2 transporters negatively regulated hepatic uptakes during acute fatty acids supply [38]. Conversely, TM4SF5 affects the internalization of CD63 (a tumor suppressor) from the PM to lysosomes [35]. Thus, TM4SF5-mediated protein complex formation with GLUT8 can transiently and dynamically affect its intracellular localization and activity.

The mechanisms underlying GLUT8 localization to PMs, as part of its transporting activity for nutritional fructose, remain unclear. GLUT8 has been shown localized mostly in lysosomes via its cytoplasmic N-terminal [D/E]XXXL[L/I] dileucine-based sorting motif [39] and to be a glucose/fructose transporter with fructose selectivity [40]. This localization and trafficking to lysosomes is mediated by the interaction of a dileucine motif with the β2-adaptin subunit of the AP-2 adaptor complex [41,42], although its trafficking from lysosomes to PMs remains unclear. As the EC2 (or LEL) and TM4 regions of TM4SF5 are crucial for its binding to GLUT8, these regions may be involved in the intracellular trafficking of GLUT8 to PMs. As lysosomes are also translocated peripherally to PMs to coordinate mTOR signaling with autophagic flux as a nutrient response [43], lysosomal TM4SF5 and GLUT8 might also be translocated peripherally to PMs, as shown in Figure 5B, with being transiently separated from each other. Furthermore, TM4SF5-mediated NFFD effects on DNL appeared to be abolished by GLUT8 suppression.

GLUT8 has recently been reported to be involved in fructose-mediated NAFLD in the female mouse liver [44]. TM4SF5 promotes NASH-associated fibrosis [17] and is involved in the development of CCl_4_-mediated fibrosis in the mouse liver [16]. The chronic and excessive intake of fructose is a dietary risk factor for NAFLD, with accompanying hyperlipidemia and insulin resistance [1]. The excessive fructose in diets can also affect other organs such as the kidney [45]. Additionally, some ingested fructose is absorbed by the intestinal enterocyte, and some fructose is transported via the portal vein to the liver, with possibly 20–30% escaping into the systemic circulation [46]. Following the absorption of ∼70% of the ingested fructose by the liver, the remainder could acutely increase 10-fold in the peripheral circulation and return to fasting levels within 2 h after fructose consumption, reaching non-hepatic organs, including the kidney [47]. Fructose in the kidney may contribute to diverse cardiometabolic risk factors, including steatosis, increased glucose production, hypertriglyceridemia, increased adiposity, and hypertension [48]. Interestingly, we observed fructose levels in the peripheral circulation and the KHK mRNA levels in the kidney increased following acute oral injection of excessive fructose, independent of TM4SF5 expression. Thus, we speculate that TM4SF5 expression may not affect fructose escaping into the kidney. Furthermore, we document that fructose fed to TM4SF5-positive or negative mice was transported comparably to their livers through the portal vein. However, the fructose taken up by hepatocytes in TM4SF5-positive mice can be catabolized by KHK, leading to the accumulations of mono-, di-, and tri-acylglycerols; these accumulations were less in *Tm4sf5*^−/−^ KO mice.

## 5 Conclusions

Taken together, our findings have revealed that excessive fructose intake-mediated steatosis under HSuD, NCFD, and/or HFFD conditions involved insulin intolerance, increased TAG, and decreased HDL accumulation in the liver, depending on TM4SF5 expression. Therefore, targeting TM4SF5 may be a promising approach to block the development of NAFLD by excessive fructose consumption.

## Author contributions

HJE, EK, and EAS designed and performed experiments; JCS, DGS, and KHL helped with analysis of lipidomics and statistics; JEK, JWJ, and YP helped with constructs and reagents; HL helped with immunofluorescence imaging; JWL designed experiments and wrote the manuscript.
