# Supplementary material for: Crosstalk between TM4SF5 and GLUT8 regulates fructose metabolism in hepatic steatosis
Source: Mol Metab. 2022 Feb 2;58:101451. doi: 10.1016/j.molmet.2022.101451 (PMC8866669; doi:10.1016/j.molmet.2022.101451)
Supplement: Supplementary file 1 [file mmc1.docx]

**Cross-talk between TM4SF5 and GLUT8 regulates fructose metabolism for hepatic steatosis in mice**

Hyejin Lee^1,2,#^, Eunmi Kim^1,2,#^, Eun-Ae Shin^1,2,#^, Jong Cheol Shon^3^, Hyunseung Sun^1,2^, Ji Eon Kim^1,2^, Jae Woo Jung^1,2^, Haesong Lee^1,2^, Yangie Pinanga^1,2^, Dae-Geun Song^4^, Kwang-Hyeon Liu^3^, and Jung Weon Lee^1,2,*^

Supplementary Figures

Figure S1


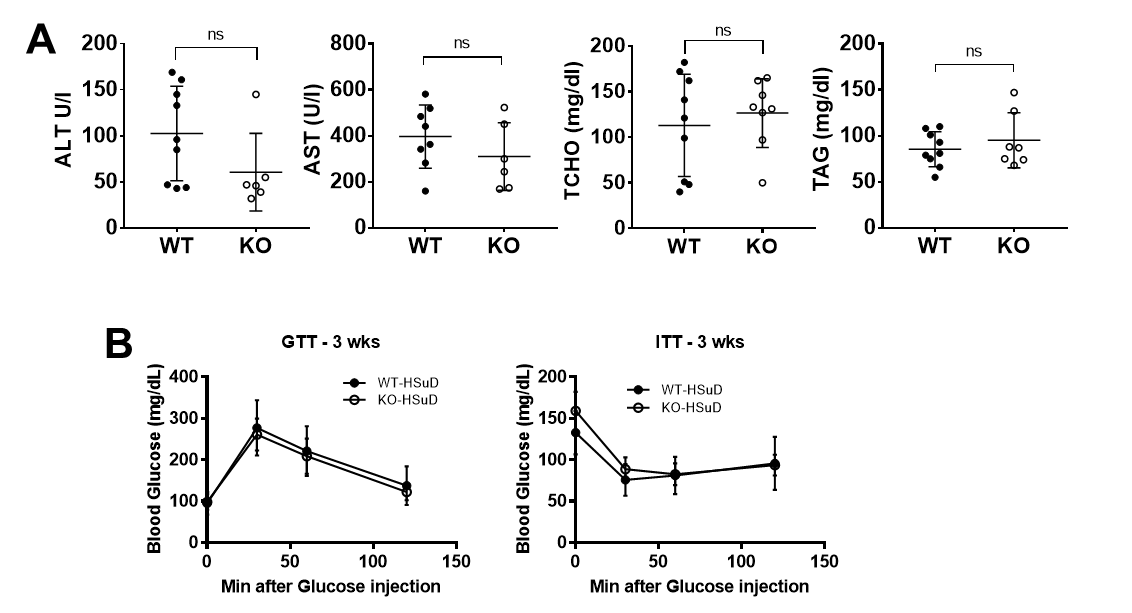


**Figure S1. No significant difference between WT and *Tm4sf5*^-/-^ KO mice fed an adjusted-calorie diet (ACD) with a high sucrose content (325 g/kg) for 3 weeks.** Six-week-old C57BL/6 WT and *Tm4sF5*^-/-^ KO male mice (n = 10) were fed an adjusted-calorie diet (ACD) with a high sucrose content (325 g/kg) for 3 weeks. (A) Blood samples from animal groups were processes for analysis for the indicated parameters. *p* values were calculated by student’s *t* test. (B) GTT and ITT were performed using intraperitoneal injections of either glucose or insulin into mice.

Figure S2


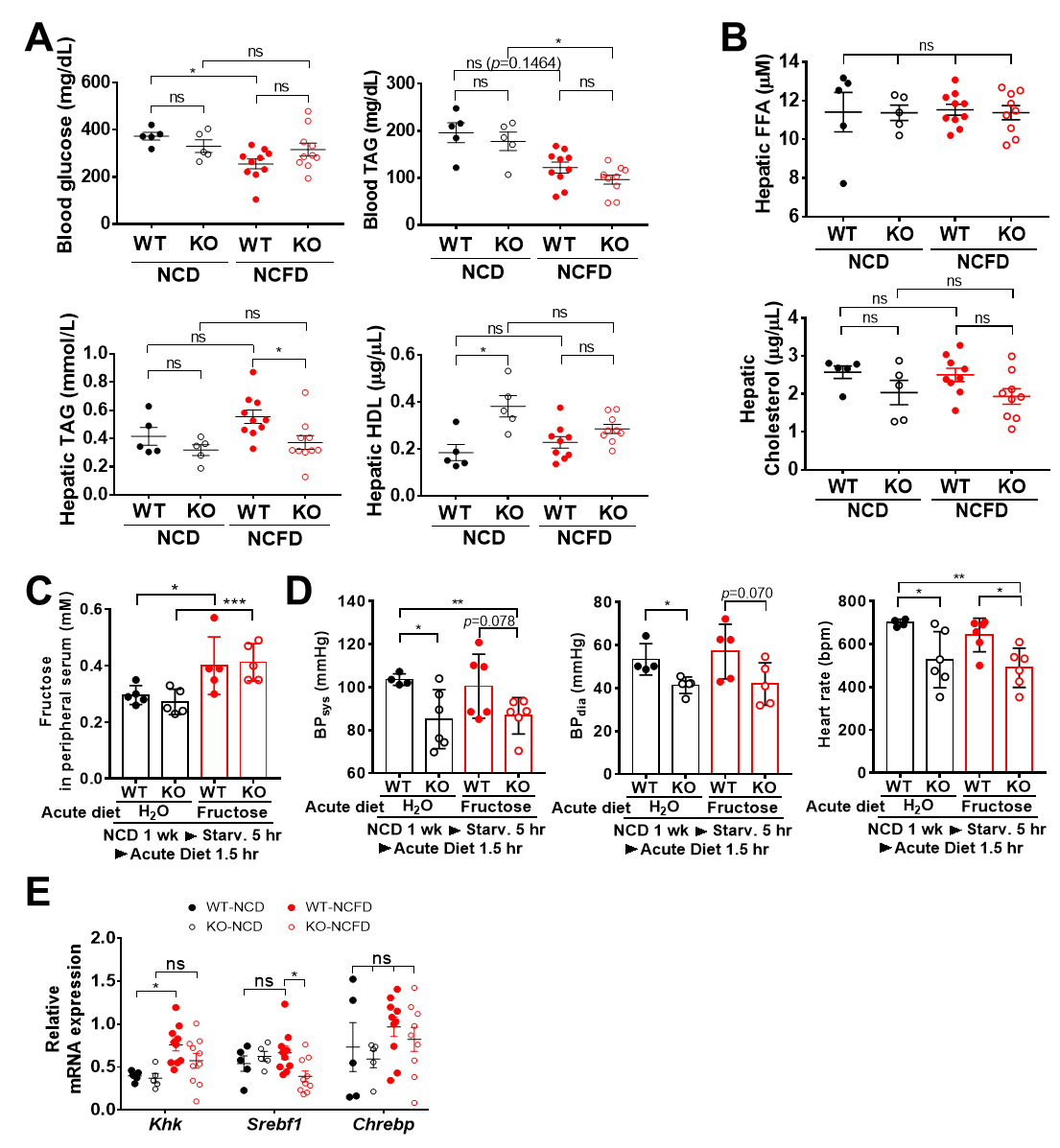


**Figure S2. Comparison of blood and hepatic lipids accumulation in WT and *Tm4sf5*^-/-^ KO mice, following NCD or NCFD for 10 weeks.** Six-week-old C56BL/6 WT and *Tm4sf5*^-/-^ KO male mice were fed *ad libitum* with either H_2_O (n=5) or fructose 30% w/v in H_2_O (NCFD, n=10) in addition to a normal chow diet (NCD) for 10 weeks. At the end of this period, the livers were collected and analyzed. (A and B) Blood and liver-tissue samples from these groups were analyzed for glucose or lipid metabolites. (C) Fructose levels were measured in the peripheral blood samples from WT or Ko mice (n=5) fed acute fructose intake (an oral injection of 4 g/kg in H_2_O). (D) Blood pressures (BP_sys_ for systolic blood pressure and BP_dia_ for diastolic blood pressure) and heart rates were measured for WT or KO mice, 1.5 hr after an (acutely) oral H_2_O or fructose injection, following NCD 1week and then 5 hr starvation. (E) Liver tissue was analyzed using qRT-PCR for the indicated mRNAs. *p* < 0.05, 0.01, or 005 values indicate statistical significance (*, **, or ***, respectively) using a one-way ANOVA. ns = not significant.

Figure S3


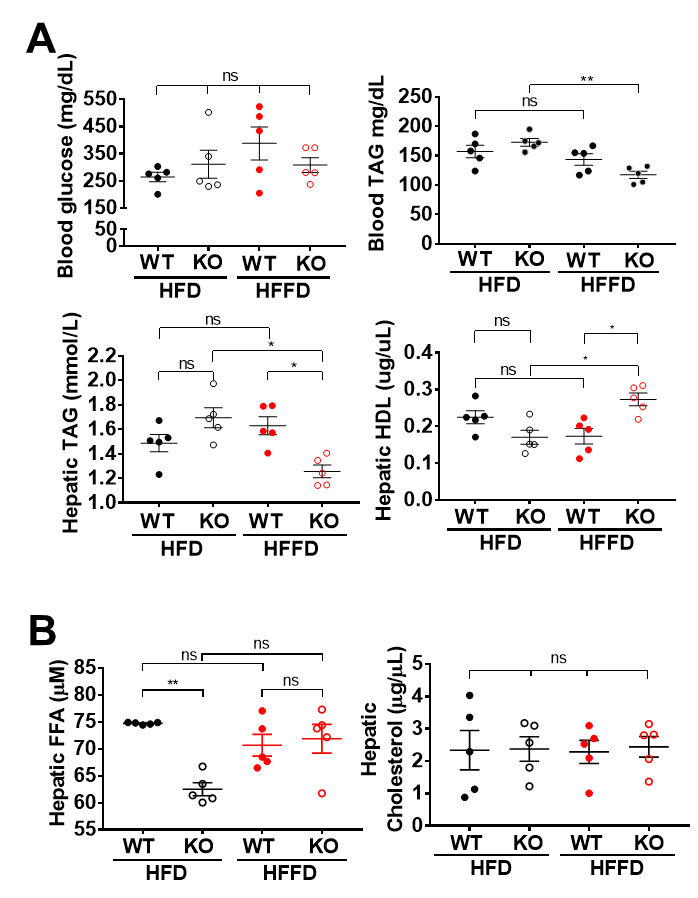


**Figure S3. Comparison of blood and hepatic lipids accumulation in WT and *Tm4sf5*^-/-^ KO mice, following HFD or HFFD for 10 weeks.** Six-week-old C56BL/6 WT and *Tm4sf5*^-/-^ KO male mice were fed *ad libitum* either high-fat diet alone (HFD, 60% kcal fat, n=5) or fructose 30% w/v in H_2_O in addition to HFD (HFFD, n=5) for 10 weeks. At the end of that period, their livers were collected and analyzed. (A and B) Blood and liver-tissue samples from the mice were analyzed for glucose or lipid metabolites. **p* < 0.05 or ***p* < 0.01 values indicate statistical significance using a one-way ANOVA. ns = not significant.

Figure S4


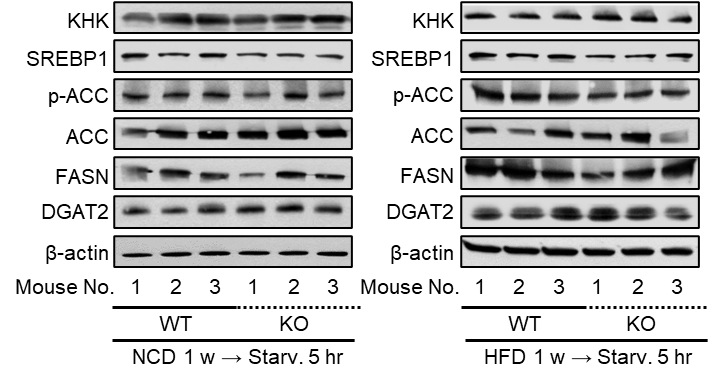


**Figure S4. No differential lipogenic enzyme expression levels in WT or *Tm4sf5*^-/-^ KO mice without acutely excessive fructose intake.** Seventeen-week-old C56BL/6 WT and *Tm4sf5*^-/-^ KO male mice [n = 5 for the H_2_O group and n = 5 for the excessive fructose (acute fructose diet) group] were fed *ad libitum* NCD or HFD for one week prior to being starved for 5 hr and then feeding with plain H_2_O. After 1.5 hr, their livers were collected and analyzed for immunoblotting for the indicated molecules.

Figure S5


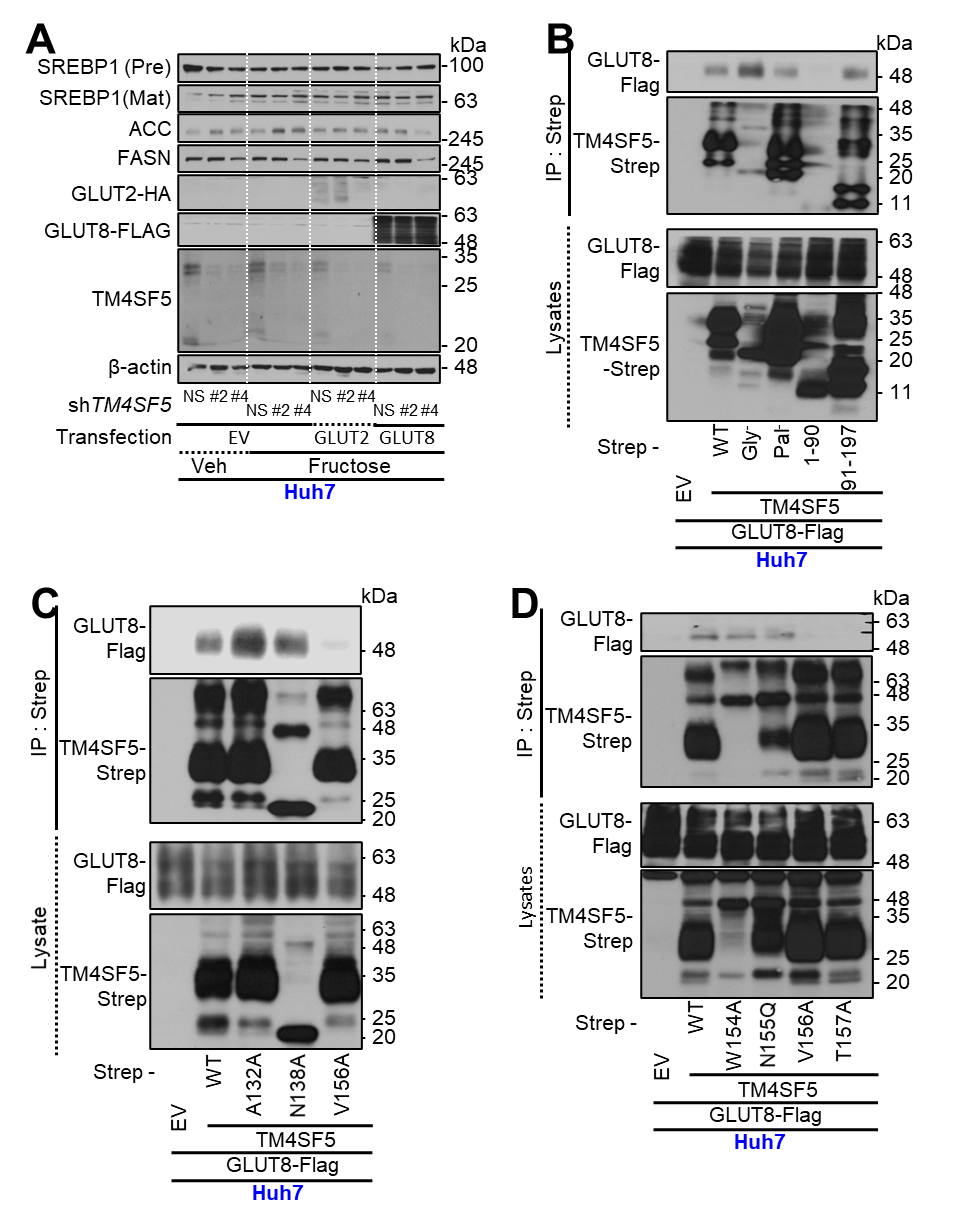


**Figure S5. Functional and physical associations between TM4SF5 and GLUT8 under excessive fructose conditions.** Endogenously TM4SF5-expressing Huh7 cells were transiently transduced with lentivirus for either a non-specific sequence (NS) or *TM4SF5* target sequences (#2 or #4) together with transfections of either empty vector (EV-FLAG), or GLUT2-HA and GLUT8-FLAG, or Strep-TM4SF5 WT or mutant plasmids for 24 hr, glucose-starved for 16 hr, and then treated with fructose (450 mg/dL) for 30 min prior to whole-cell lysate preparation for standard western-blot analysis (A) or co-pulldown experiments using streptavidin-conjugated agarose beads, for standard western-blot analysis of the indicated molecules (B-D). The data shown represent three independent experiments.
